# Magnetometer-Augmented IMU Simulator: In-Depth Elaboration

**DOI:** 10.3390/s150305293

**Published:** 2015-03-04

**Authors:** Thomas Brunner, Jean-Philippe Lauffenburger, Sébastien Changey, Michel Basset

**Affiliations:** 1 French-German Research Institute of Saint-Louis (ISL, Guidance, Navigation and Control (GNC) Department), 5 rue du Général Cassagnou, Saint-Louis 68300, France; E-Mail: sebastien.changey@isl.eu; 2 Laboratoire MIPS - EA2332, Université de Haute-Alsace, 12 rue des Frères Lumière, Mulhouse Cedex 68093, France; E-Mails: jean-philippe.lauffenburger@uha.fr (J.-P.L.); michel.basset@uha.fr (M.B.)

**Keywords:** simulator, magnetometer, absolute magnetic reference, IMU, modeling

## Abstract

The location of objects is a growing research topic due, for instance, to the expansion of civil drones or intelligent vehicles. This expansion was made possible through the development of microelectromechanical systems (MEMS), inexpensive and miniaturized inertial sensors. In this context, this article describes the development of a new simulator which generates sensor measurements, giving a specific input trajectory. This will allow the comparison of pose estimation algorithms. To develop this simulator, the measurement equations of every type of sensor have to be analytically determined. To achieve this objective, classical kinematic equations are used for the more common sensors, *i.e.*, accelerometers and rate gyroscopes. As nowadays, the MEMS inertial measurement units (IMUs) are generally magnetometer-augmented, an absolute world magnetic model is implemented. After the determination of the perfect measurement (through the error-free sensor models), realistic error models are developed to simulate real IMU behavior. Finally, the developed simulator is subjected to different validation tests.

## 1. Introduction

The dynamic location of objects is a predominant research topic in many fields (robotics [1], intelligent vehicles [2], UAVs [3], bio-logging [4], *etc.*). One of the main techniques for precise object location is inertial navigation. Inertial sensors exploit proprioceptive measurements, which result from the evolution of the object's position. The combination of different proprioceptive sensors (accelerometers and rate gyroscopes) is known as an Inertial Measurement Unit (IMU) [5]. With the rapid development of MEMS (microelectromechanical systems) technology, low-cost sensors are now common, offering more diversity in the application fields of inertial navigation.

As this domain is expanding, some applications require the sensors to withstand high dynamic loads; others are just embedded in our pockets; smartphones, for example, use MEMS technology for some navigation purposes. In the latter case, magnetometers are also added to measure the magnetic field and to determine the heading of the phone.

Another well-known method to perform navigation is the use of satellite positioning systems (*i.e.*, Global Navigation Satellite Systems (GNSS)), which directly provide the position of a mobile device using satellite-transmitted signals. Since the signals are not always available (such as indoor or underwater navigation, for example), GNSS is not considered here.

In general, estimation algorithms based on classical IMUs (based on accelerometers and gyroscopes) are developed for a given application, incorporating the dynamic model of the system to increase the algorithm knowledge and to improve precision and accuracy. Unfortunately, this generally implies poor results when the estimation algorithm is used on an application with different dynamics. The idea is to develop estimation algorithms that can be more generic (application and system independent), robust and with a high level of integrity. In this case, the dynamic model has to be removed, causing a lack of information. Redundant information is then required to perform data fusion in order to correct the skewed sensor data. As in the previous example of the smartphone, in the context of this work, classical IMUs are augmented with magnetic sensors.

To test these algorithms in different situations, a simulator is built. This allows the evaluation of algorithms for a known and fully controlled input trajectory describing the movement of any kind of object, ranging from low dynamic systems, like animals, to high dynamic ones, like flying objects. The aim is then to simulate every kind of trajectory: long-range, as well as short-range displacements. A number of usual hypothesis, like flat Earth or non-rotating reference frames, cannot be maintained in this context.

**Figure 1.**
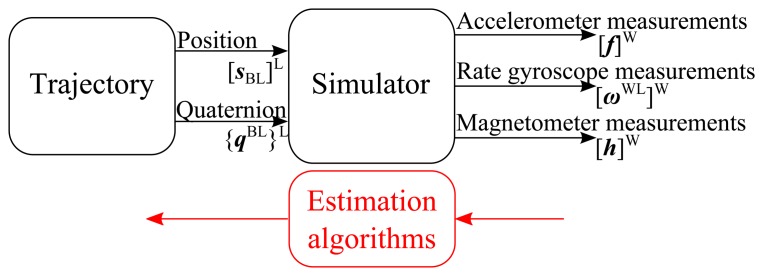
Simulator principle.

This paper presents the modeling of a magnetometer-augmented IMU. A MATLAB simulator based on the kinematic models of the sensors (accelerometers, rate gyroscopes and magnetometers) is proposed. To achieve this modeling, different characteristics are taken into account: the multiple reference frames required to deal with short- and long-distance trajectories (inertial frame, body frame, *etc.*); the location of the IMU, which can be different from the center of the object; the modeling of the accelerometers, rate gyroscopes and magnetometers, which also takes into account several sensor imperfections (bias, noise, *etc.*). Finally, this tool is designed to provide sensor measurements, from an input trajectory defined by the object orientation quaternion and its spatial position (see Figure 1).

Moreover, additional parameters, like the position of the IMU with respect to the object, allow simulating measurements of the sensors for the same trajectory in different configurations. The investigation of multiple IMU systems and complex architectures [6–8] can thus be studied.

Other simulators already exist in the literature: In [9], the focus is on the error models for the skewed sensors. Indeed, the measurements are not determined from an input trajectory; the error-free accelerations and rotational speeds are the input of the cited simulator. The outputs then consist of the skewed measurements. In [10], a simulator is built for different applications: to perform and test calibration processes and to validate new mathematical models involving IMUs. Unfortunately, magnetometers are not considered. The validation processes are also generally simple: for the error-free signals, only a static validation is performed. Meanwhile, the paper shows interesting results for sensor error modeling, comparing Allan Variance results.

This paper shows a more accurate and complete validation of a magnetometer-augmented IMU simulator than the previously cited papers. It consists of placing an IMU in a three-axis table, which has been developed at ISL (French-German Research Institute of Saint-Louis)[11]. The absolute encoders of the electric engines are used to measure the 3D motion of the table. To validate the simulator that is even more dynamic in the trajectory, another testing method is proposed, using a validated high dynamic trajectory simulator.

The outline of the paper is as follows: Section 2 describes the principle and the tools needed to build the simulator, and Section 3 develops the error-free equations of the measurements. In Section 4, the sensor error modeling is described. Section 5 shows the simulator validation process, and Section 6 concludes the paper.

## 2. Modeling of the IMU Measurements

### 2.1. Principle

In this work, a three-axis IMU will be considered, composed of three accelerometers, three rate gyroscopes and, finally, three magnetometers. As mentioned earlier, the aim of the simulator is to compute all of the sensor measurements from the IMU according to a measured or an *a priori* known object trajectory. The principle of the simulator is shown in Figure 1.

From a trajectory describing the evolution of the spatial position [***s***_BL_]^L^ (the notation rules are described in Section 2.2 for clarity's sake) and the angular position with respect to a frame L (using the quaternion {***q***^BL^}^L^), the simulator determines the accelerations, the rotational speeds and the magnetic measurements of the IMU sensors in the IMU frame W. Thus, the simulator performs an inverse modeling of the object dynamics taking account the sensor imperfections, such as drifts, biases, *etc.* (see Section 4). Its main purpose is the validation of motion estimation algorithms by comparing the input trajectory to the estimated one. This trajectory may correspond to a real or simulated motion allowing the estimation algorithms to be tested under the desired conditions. By adding some information about the plant, it is also possible to test different positions and orientations of the IMU with respect to the object. This can be used for redundant IMU systems, as well [12].

### 2.2. Notations

This section specifies the notation rules used to develop the kinematic model of each sensor. The notation is mainly inspired by [13] and is detailed in Table 1. For the kinematic equations, the subscripts refer to a point, and the exponents specify the reference frame.

### 2.3. Frames and Coordinate Systems

**Figure 2.**
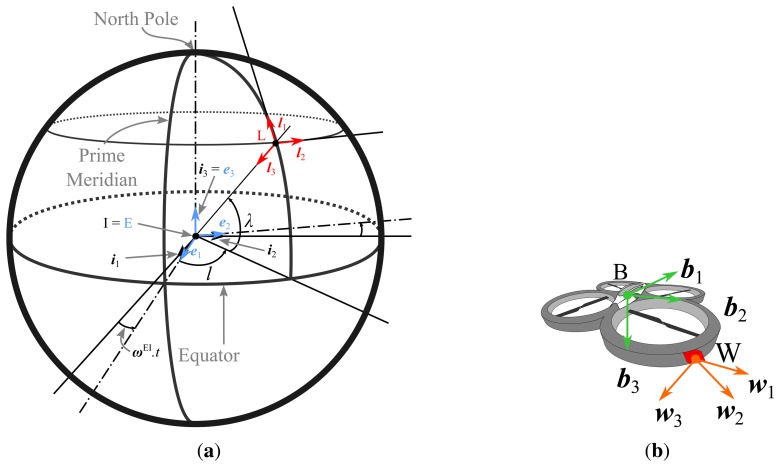
Illustration of the different frames: inertial frame (**I**), Earth frame (**E**), local frame (**L**), body frame (**B**) and work frame (**W**).(**a**) Inertial, Earth and local frames; (**b**) Body and work frames.

Numerous reference frames are employed in navigation [13,14]. In this work, the following frames will be considered (see Figure 2):

- The inertial frame (I) is centered on the Earth. Its third axis is directed to the North Pole. The other axes are in the equatorial plane. This frame does not rotate with the Earth; therefore, it is considered to be the inertial reference. To simplify, the inertial frame has the same axes as the Earth's frame (E) at time *t* = 0.
- The Earth's frame (E) is centered on the Earth and follows the Earth's rotation (constant speed *ω*^EI^), unlike (I) . Its first axis is directed to the prime meridian in the equatorial plane; the third axis is directed to the North Pole; and the second completes the orthogonal basis.
- The local coordinate system (L) is located at the center of gravity of the object. Its orientation is tangent to the surface of the Earth and can be obtained by means of its position with respect to (E) described by two angles: the latitude (*λ*) and the longitude (*l*). The Earth, inertial and local frames are illustrated in Figure 2a.
- The body frame (B) is related to the object orientation (see Figure 2b). Its orientation with respect to (L) is given by the classical Euler angles.
- The IMU frame (W) can be misaligned with the body frame. However, (W) is fixed with respect to (B) and is dependent on its location on the object.

Any axis of a frame is named with the lower-case of the frame name and a subscript giving its number, such that the three vectors of frame (E) are then ***e***_1_, ***e***_2_ and ***e***_3_ (see Figure 2).

The use of the inertial and Earth's frames is justified by our choice to simulate long-range or long-term trajectories. The classical approximation of the flat Earth has then to be removed, and the Earth is no longer an inertial frame, as it rotates.

### 2.4. Quaternions

#### 2.4.1. Definition

The object orientation can be expressed in multiple ways: Euler angles, quaternion, vector and angle, *etc.* The solution selected in sensor modeling work is the quaternion [5]. Quaternions avoid the main problem of Euler angles: the gimbals lock, which gives some numerical issues for a number of known orientations. This decision results from the need to have a generic simulation tool performing all kinds of displacements. In this case, having forbidden orientations is a problem and unacceptable. The notions necessary for the understanding of this paper are briefly given in this section. Readers who would like to go into more details can refer to [15].

Introduced by Hamilton, quaternions are hypercomplex numbers with four components: a scalar *q*_0_ and three imaginary numbers, *q*_1_, *q*_2_ and *q*_3_. One quaternion ***q*** can be expressed as:

(1) $$q=q_{0}+q_{1}i+q_{2}j+q_{3}k$$

with vectors ***i***, ***j*** and ***k*** describing an orthonormal basis. A pure quaternion is a quaternion with a scalar part *q*_0_ equal to zero.

Another way of representing quaternions is to regroup the complex numbers in a vector ***q̄***_v_ = [*q*_1_ *q*_2_ *q*_3_]. To avoid confusion between vectors and quaternions, brackets {} are used instead of [].

(2) $$\text{q}=\left\{\begin{array}{c} {\text{q}}_{0}\\ q_{\text{v}} \end{array}\right\}$$

Using this notation, the multiplication between two quaternions, written ***r*** = ***p*** ⊗ ***q***, gives:

(3) $$r=\left\{\begin{array}{c} {\text{p}}_{0}{\text{q}}_{0}-p_{\text{v}}\cdot q_{\text{v}}\\ {\text{p}}_{0}q_{\text{v}}+q_{0}p_{\text{v}}+p_{\text{v}}\wedge q_{\text{V}} \end{array}\right\}$$

#### 2.4.2. Quaternions and Rotations

The complex part of the quaternion (***q***_v_) is equivalent to a 3D vector. It can be very helpful to represent rotations: a rotation around an axis ***d*** by an angle *α* can be represented [2] by a unit quaternion (its norm is equal to one)

(4) $$q=\underset{q_{0}}{\underset{︸}{\cos\frac{\alpha }{2}}}+\underset{q_{\text{v}}}{\underset{︸}{\sin\frac{\alpha }{2}d}}$$

As any combination of rotations can be, in the end, expressed by one unique rotation, quaternions can then express any complex rotation from one frame to another.

The image ***x′*** of a vector ***x*** by a rotation represented by ***q*** can be obtained by the following quaternion product:

(5) $$x^{\prime }=q\;⊗\;x\;⊗\;\tilde{q}$$

where ***q̃***, the conjugate of ***q***, is defined as ***q̃*** = *q*_0_ − ***q***_v_. In Equation (5), the vectors ***x*** and ***x′*** are considered to be pure quaternions, with their components *x*_0_ and $x_{0}^{\prime }=0$.

Another important characteristic of unit-norm quaternions is the product of a quaternion and its conjugate:

(6) $$q⊗\tilde{q}=\left\{\begin{array}{c} 1\\ 0 \end{array}\right\}$$

A quaternion expressing the orientation of a frame A with respect to frame B and with the complex part expressed in the C coordinate system is written {***q***^AB^}^C^.

## 3. Error-free Inertial Sensor Modeling

In this section, the way of deriving the sensor measurements from the trajectory information is described considering perfect sensors, *i.e.*, without any errors. The imperfections are described and added in the next section. The final objective of this research is to obtain pose observers , taking into account the absolute magnetic reference data. That is why particular interest will be given on the magnetic field modeling and magnetometer measurement estimation. The objective is also to simulate any kind of trajectory: short *vs.* long, high *vs.* low dynamics, *etc.* This section competes a description provided in a previous paper by the present authors [11].

### 3.1. Rate Gyroscopes

A rate gyroscope measures the rotational speed of the sensor around its sensitive axis with respect to (I). The measurement is then expressed in the sensor coordinate system (W). It can be determined from the quaternion and its derivative [15]:

(7) $${\left\{{\dot{q}}^{\text{WI}}\right\}}^{\text{I}}=\frac{1}{2}{\left\{q^{\text{WI}}\right\}}^{\text{I}}⊗\;{\left\{\;\omega^{\text{WI}}\right\}}^{\text{W}}$$

***q***^WI^Quaternion expressing the orientation of frame W with respect to I***q̇***^WI^Time derivative of ***q***^WI^***ω***^WI^Pure quaternion formed from the rotational speed vector of (W) with respect to (I)


As described by Equation (6), ***q̃*** ⊗ ***q*** = **1**, thus multiplying (7) on the left by {***q̃***^WI^}^I^ gives:

(8) $${\left\{\omega^{\text{WI}}\right\}}^{\text{W}}=2{\left\{{\tilde{q}}^{\text{WI}}\right\}}^{\text{I}}\;⊗\;{\left\{{\dot{q}}^{\text{WI}}\right\}}^{\text{I}}$$

The last step is to determine {***q***^WI^}^I^ , such that:

(9) $${\left\{q^{\text{WI}}\right\}}^{\text{I}}={\left\{q^{\text{WB}}\right\}}^{\text{I}}\;⊗\;{\left\{q^{\text{BL}}\right\}}^{\text{I}}\;⊗\;{\left\{q^{\text{LE}}\right\}}^{\text{I}}\;⊗\;{\left\{q^{\text{EI}}\right\}}^{\text{I}}$$

Then, {***q̇***^WI^}^I^ is numerically calculated to finally obtain {***ω*^WI^**}^W^. Its vector part $\omega_{\text{v}}^{\text{WI}}$ is expressed in (W) and is composed of the three rotational speeds, measured by each sensor.

### 3.2. Accelerometers

An accelerometer measures the specific force applied along its main axis (in frame W) [13]. The specific force is defined by the sum of all non-gravitational forces applied on the object divided by the object mass. It is thus the absolute acceleration of the object ( $a_{W}^{\text{I}}$) minus the gravitational field, such that:

(10) $${\left[f\right]}^{\text{W}}={\left[a_{W}^{\text{I}}\right]}^{\text{W}}-{\left[g_{\text{f}}\right]}^{\text{W}}$$

***f***Specific force measured by the accelerometers
$a_{W}^{\text{I}}$Acceleration of point W with respect to frame I***g***_f_Gravity field applied to the three-axis accelerometer


As a reminder, the gravitational field is expressed as [13]:

(11) $$g_{\text{f}}=g+a_{e}=-GM\frac{s_{\text{WE}}}{{\left\|s_{\text{WE}}\right\|}^{3}}-\omega^{\text{EI}}\wedge \left(\omega^{\text{EI}}\wedge s_{\text{WE}}\right)$$

***g***Gravity force***a****_e_*Acceleration due to the rotation of the Earth*G*Gravitational constant*M*Mass of the Earth*s*_WE_Position of the sensors with respect to (E)*ω*^EI^Rotational speed of the Earth around its axis


The objective is then to determine the expression of [***f***]^W^ from the evolution of the object position [***s***_BL_]^L^, which is an input of the simulator. However, the sensors are not positioned at the center of gravity of the object. Consequently, deriving the position of the object will not lead to the acceleration of the IMU, but to that of the object. After transferring the input position [***s***_BL_]^L^ in frame (E), the equation of the total acceleration applied to the IMU is obtained by the following derivation process (for the sake of clarity, it is assumed that all variables are expressed in the same coordinate system; the brackets [ ]^X^ are then left out) [13]:

(12) $$\begin{array}{ll} \upsilon_{\text{W}}^{\text{I}} & ={\frac{ds_{\text{WI}}}{dt}|}_{\text{I}}\\ & ={\frac{ds_{\text{WI}}}{dt}|}_{\text{E}}+\omega^{\text{EI}}\wedge s_{\text{WI}}\\ & ={\frac{ds_{\text{WB}}}{dt}|}_{\text{E}}+{\frac{ds_{\text{BE}}}{dt}|}_{\text{E}}+\omega^{\text{EI}}\wedge s_{\text{WI}}\\ & =\upsilon_{B}^{\text{E}}+{\frac{ds_{\text{WB}}}{dt}|}_{\text{B}}+\omega^{\text{BE}}\wedge {\text{s}}_{\text{WB}}+\omega^{\text{EI}}\wedge s_{\text{WI}} \end{array}$$

(13) $$\upsilon_{W}^{\text{I}}=\upsilon_{B}^{\text{E}}+\omega^{\text{BE}}\wedge s_{\text{WB}}+\omega^{\text{EI}}\wedge s_{\text{WI}}$$

and:

(14) $$a_{\text{W}}^{I}={\frac{d\upsilon_{W}^{\text{I}}}{dt}|}_{\text{I}}$$

(15) $$\begin{array}{ll} a_{W}^{\text{I}} & =\overset{I}{\overset{︷}{a_{B}^{\text{E}}}}+\overset{II}{\overset{︷}{{\frac{d\omega^{\text{BE}}}{dt}|}_{\text{B}}\wedge s_{\text{WB}}+\omega^{\text{BE}}\wedge \left(\omega^{\text{BE}}\wedge s_{\text{WB}}\right)}}\\ & +\underset{III}{\underset{︸}{\omega^{\text{EI}}\wedge \left(2\left(\upsilon_{B}^{\text{E}}+\omega^{\text{BE}}\wedge s_{\text{WB}}\right)+\omega^{\text{EI}}\wedge s_{\text{WI}}\right)}} \end{array}$$

$\upsilon_{W}^{\text{I}}$Speed of point W with respect to frame I
$a_{B}^{\text{E}}$Acceleration of point B with respect to frame E


In Equation (15), the acceleration can be divided into three parts: Part **I** is the acceleration of the object with respect to the Earth, *i.e.*, the acceleration obtained from the input position of the simulator. Part **II** is the acceleration due to the position difference between the IMU and the object center of gravity. By changing the value of ***s***_WB_ in the input, it is possible to simulate different IMU positions with respect to the object for the same trajectory. Part **III** is the acceleration caused by the Earth's rotation around its axis. These three parts are essential to the simulator: the first part is obviously needed; the second part is very important as it expresses the different positions of the IMU with respect to the object (multi-IMU applications rely on this part); and the last part is needed for long-time trajectories.

Finally, the specific force can be obtained by subtracting Equation (11) from Equation (15).

### 3.3. Magnetometers

A three-axis magnetometer measures the direction and the intensity of the magnetic field around the sensor. If this magnetic field is not perturbed, it corresponds to the Earth's magnetic field. The magnetometer measurements are the projection of this magnetic field ***h*** in the frame (W):

(16) $${\left[h\right]}^{\text{W}}={\left[T\right]}^{\text{WL}}{\left[h\right]}^{\text{L}}$$

The Earth's magnetic field is (in the Northern Hemisphere) directed toward the North Magnetic Pole and the inside of the Earth. The difference in position between the North Magnetic Pole and Geographic North Poles leads to a non-zero component on the *l*_2_ axis. The knowledge of the reference value of the magnetic field is then primordial for the heading correction of the different pose estimators. With the aim of simulating all kinds of trajectories, an Earth's magnetic field model, called the World Magnetic Model [16], has been implemented in the simulator to provide the reference unperturbed magnetic value for any object position. This model allows also simulating any movement between the two polar circles. The value of [***h***]^L^ is given by this model.

This model was obtained by the interpolation of multiple measurement centers all on the Globe and is an empirical model. This is why it is only valid for five years and is constantly evolving. The implementation of this model also provides the value of the reference everywhere on the surface of the Earth. This allows simulations of trajectories everywhere, but also simulations of a long duration.

## 4. Inertial Sensor Error Modeling

Now that the hypothetical sensor measurements are determined, the imperfections have to be added to produce realistic sensor outputs. The outputs of the simulator are considered to be calibrated measurements, *i.e.*, the misalignment and scale factors are assumed to be corrected. The considered sensor errors are biases, sensor dynamics and multiple types of noises. Thus, the output of a sensor ***c****_m_* is modified compared to the errorless value ***c*** introduced in Section 3. ***c****_m_* can be expressed by adding the error sources:

(17) $$c_{\text{m}}=K*c+b+w$$

with ***K*** the indicating transfer function describing the sensor dynamics, ***b*** the bias and ***w*** the sensor noise.

The noise ***w*** is a white noise. The biases have a constant part, but can evolve during a trajectory. This evolution is mainly due to random walk and correlated noises [17].

### 4.1. Sensor Dynamics and Bandwidth

The first modeled imperfection is the dynamics of the sensor. In this work, a first-order transfer function is used to reproduce the main characteristic, which is the bandwidth. The transfer function considered is:

(18) $$K(s)=\frac{\omega_{c}}{s+\omega_{c}}$$

with *ω_c_* denoting the cut-off pulsation at −3 dB, which can be read on the sensor datasheet. It is a user-defined parameter in the simulator configuration.

### 4.2. Bias Modeling

The second type of imperfection is the bias. It can be split into two parts: a constant one (***b***_cst_) and a time-evolving one (***b***_ev_). The constant part can be corrected during a calibration process, but some residuals can remain.

To model the bias evolution, two processes are used, as suggested in [17]; a first-order Gauss-Markov process (***b***_gm_) and a random walk (***b***_rw_). The first is used to represent a wide number of physical processes. It is an autoregressive process with a correlation time *T_c_*. The parameters needed are the correlation time and the standard deviation *σ*_gm_ of the noise. The random walk, on the other hand, is the result of the integration of a white noise. The standard deviation *σ*_rw_ of the integrated noise has to be specified in the parameters of the simulator. Finally, the evolving part of the bias is given by:

(19) $${\left[\begin{array}{c} b_{\text{gm}}\\ b_{\text{rw}} \end{array}\right]}_{k}=\left[\begin{array}{cc} \left(1-\frac{\Delta t}{T_{c}}\right) & 0\\ 0 & 1 \end{array}\right]\;{\left[\begin{array}{c} b_{\text{gm}}\\ b_{\text{rw}} \end{array}\right]}_{k-1}+\left[\begin{array}{c} \sigma_{\text{gm}}\sqrt{\left(1-\exp\left(-2\frac{\Delta t}{T_{c}}\right)\right)}\\ \sigma_{\text{rw}}\sqrt{\Delta t} \end{array}\right]\;u_{k}$$

(20) $$b_{\text{ev}}=\left[\begin{array}{cc} 1 & 1 \end{array}\right]\;{\left[\begin{array}{c} b_{\text{gm}}\\ b_{\text{rw}} \end{array}\right]}_{k}$$

with ***u****_k_* a unitary white noise.

The last implemented process error is ***w***, the error due to the white noise. This noise is considered to be zero-mean and Gaussian. The last parameter to be specified is then its standard deviation.

### 4.3. Noise Parameter Identification

**Figure 3.**
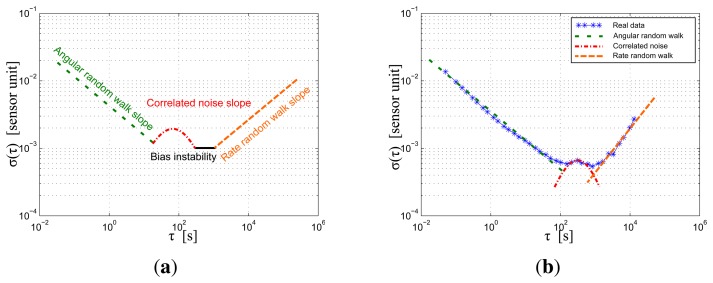
Allan variance sketch and its application to the considered IMU. (**a**) Sketch of the Allan variance process. Visible imperfections are described; (**b**) Application of the Allan variance on one accelerometer.

To simulate a commercially available low-cost IMU, multiple experiments were conducted on one IMU. The Allan variance was employed as suggested by [18,19] to identify the noise parameters. It is a widely-used time-domain technique in the modeling of inertial sensor errors, which is able to determine long-term noises. To perform this approach, the IMU has to remain completely still during several hours. Figure 3 shows a typical Allan variance plot and its interpretation. The values are extracted from the measurements as explained in [19]. Figure 3a is an hypothetical Allan variance sketch, where the typical noise components are described. The angular random walk slope illustrates the effect of a white noise. The rate random walk slope is the result of a random walk noise (*cf.* the previous section). The correlation noise slope comes from the correlated part of the measurements, and finally, the bias instability is due to multiple physical noises. As the variance is a function of the time cluster size, it is possible to interpret the influence of each type of noise: the angular random walk noise is a problem in short time, but can be reduced by averaging the measurements, while the rate random walk noise affects more the long-term measurements more. Figure 3b is the result of this calculation on one of the accelerometer of the IMU. This figure has been made with a 12-hour measurement during which the IMU stayed motionless. By comparing the two figures, it is clear that not every component of Figure 3a is present in Figure 3b. This is the main reason why the bias evolution was simulated by superposing of a correlated noise and a random walk process.

As an example, Table 2 shows the results of this procedure for an IMU from SBG Systems [20]. It gathers all the necessary data for the simulation of the IMU imperfections in the simulator.

## 5. Simulator Validation

After implementing the Equations (8) and (15)–(17) in MATLAB, a validation process is carried out. The first part is dedicated to the validation of the kinematic equations. For this purpose, a simulation is performed with a known trajectory and without imperfections. In the second part, the error model is validated by comparing Allan variances from simulated and real measurements.

### 5.1. Kinematic Validation

**Figure 4.**
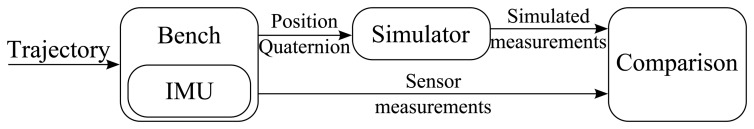
Simulator validation sketch.

The validation principle is illustrated in Figure 4. An IMU is subject to a trajectory which is perfectly known or measured. The trajectory information (position and rotation quaternion) is used as the input of the simulator, and a comparison is performed between the output of the simulator and the recorded measurements. In the following, two types of validation are performed: hardware and software. The hardware validation uses a real IMU to test the simulator output against real measurements. The drawback here is the limited dynamics in the trajectory due to the employed test bed. Thus, a software validation with a validated projectile simulator is performed in a second step.

#### 5.1.1. Test Bed Validation

**Figure 5.**
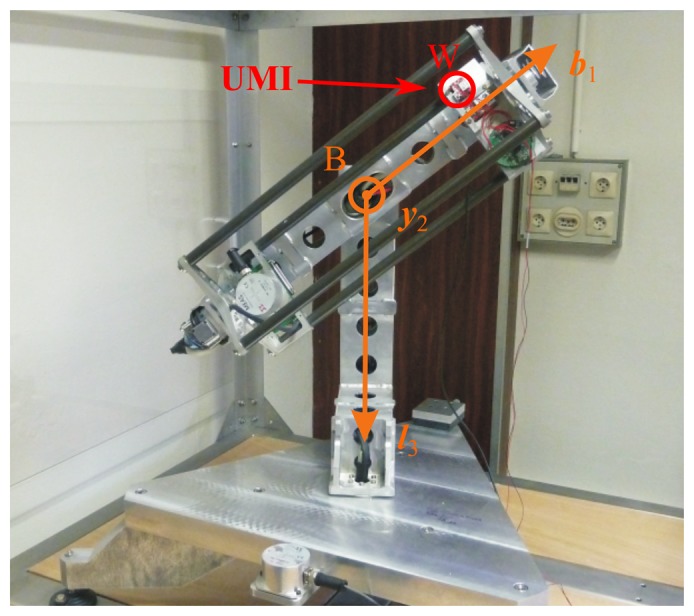
Test bed: three-axis table.

For the hardware validation, the 3D test bench from ISL is used (see Figure 5). This first validation process allows us to test our simulations against real data. Using this three-degrees-of-freedom table, measurements of the angles are very accurate (< 0.1°) and are obtained by the motor coders. The IMU position with respect to the table impacts the acceleration measurements. If this position is different from the center of rotation, accelerations due to the rotation can be measured instead of only the gravitational force projection alone. For this validation, the rotational table is considered as the body frame; the position and the orientation of W with respect to B has to be placed as a parameter in the simulator.

**Figure 6.**
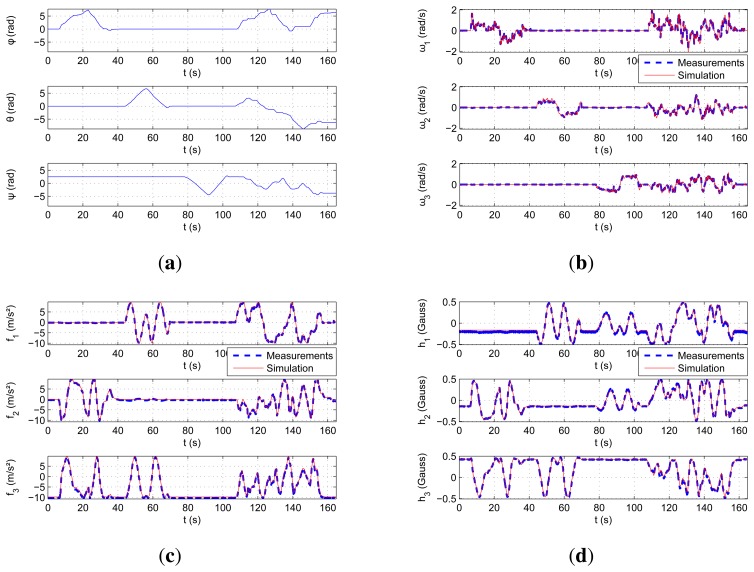
Plot of the trajectory and the different comparisons of the measurements. (**a**) Euler angles describing the movement of the 3D-table; (**b**) comparison of the measured and simulated rotational speeds; (**c**) comparison of the measured and simulated specific forces; (**d**) comparison of the measured and simulated magnetometer measurements.

The performed motion is characterized by the Euler angles illustrated in Figure 6a. This motion is divided into two parts. The first part, up to *t* = 100 s, is composed of three pure rotations around each axis separately; the second part of the movement is an arbitrary rotation around the three axes simultaneously. The measurement comparisons of each type of sensor are shown in Figure 6b–d. It can be seen that the simulated measurements are very close to the real ones.

The overall performance of the simulator is shown in the first line of Table 3. The normalized root mean square error (NRMSE) of the accelerometer measurements are 2.5% at maximum. The rate gyroscopes display an error below 1.5%, which represents around 6°/s. The magnetometers also show errors of less than 3.7%. Even if these quantities seem to be high, its a comparison to real data from a low-cost IMU, which means that the measurements include a lot of noise and imperfections.

**Figure 7.**
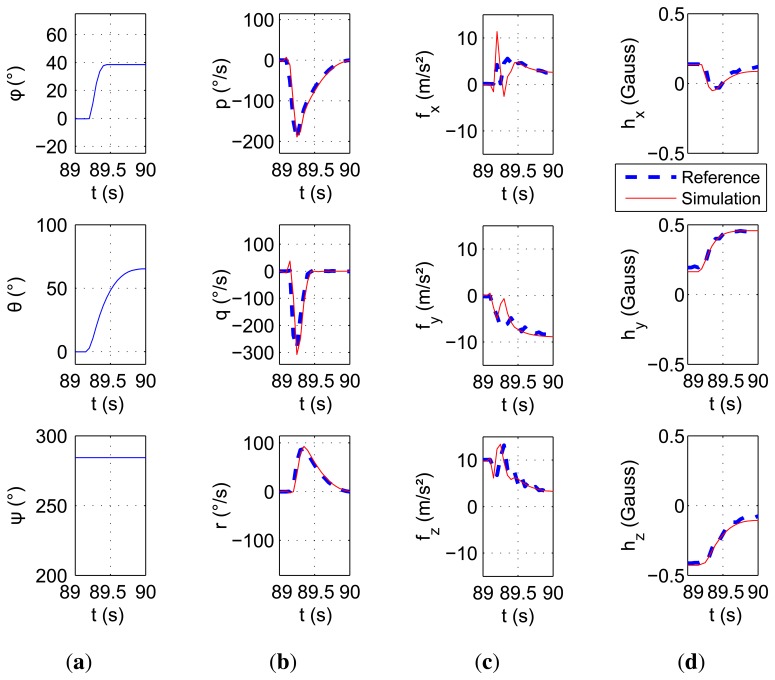
Simulation of a fast motion. (**a**) Euler angles; (**b**) angle rates; (**c**) specific force; (**d**) magnetic field.

While treating another data series, a quick and short motion has been recorded. This movement is illustrated in Figure 7a. The bench was able to measure the motion correctly, and results are well observed and reproduced by the simulator. This quick motion shows that if the input is precise enough, the dynamics can be well reproduced.

#### 5.1.2. Cross-Simulation Validation

The purpose of the second validation is here to endorse the simulator. It follows the same procedure as the previous validation. The difference lies in the use of another simulator instead of the 3D table. This second simulator deals with a high-dynamics application: gun-launched projectile. This simulator was validated by many real projectile shots [21].

**Figure 8.**
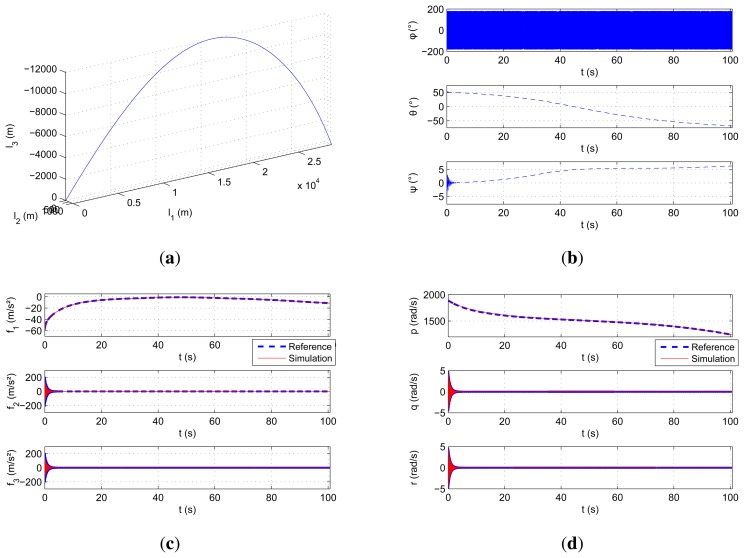
Validation plots thanks to the projectile simulator. (**a**) 3D trajectory of the gun-launched projectile; (**b**) Euler angles describing the orientation of the gyro-stabilized projectile. The stabilizing spin is around the first axis; (**c**) Comparison of the specific forces; (**d**) comparison of the rotational speeds.

For the validation process, the performed motion is illustrated in Figure 8a,b. It is the simulation of a 155-mm caliber shell. The sensors are placed at two calibers from the center of mass of the shell. As shown, the motion is around 30 km long, and the linear and rotational speeds can exceed 900 m/s and 300 Hz (≃ 1800 rad/s), respectively, on the main axes.

The results are illustrated in Figure 8c,d. Unfortunately, the magnetometer data are not integrated in the projectile simulator.

As it is an uncontrolled trajectory, oscillations keep occurring all along the way. Thus, vibrations can be seen on radial accelerations and rate gyroscopes. Table 3 (second raw) shows the similarities between the curves. The values of the errors and standard-deviations are even lower than for the test bed validations. This is due to the projectile simulator, which also considers perfect sensors and provides error-free measurements. The equations are then validated even for high-dynamics.

### 5.2. Error Model Validation

This section is dedicated to the error model validation and, precisely, the implemented stochastic model. For this, the Allan variance is used as follows: multiple simulations of a static IMU are executed, and the Allan variance is inferred on each measurement. Then, the results are compared to an Allan variance applied to real measurements.

**Figure 9.**
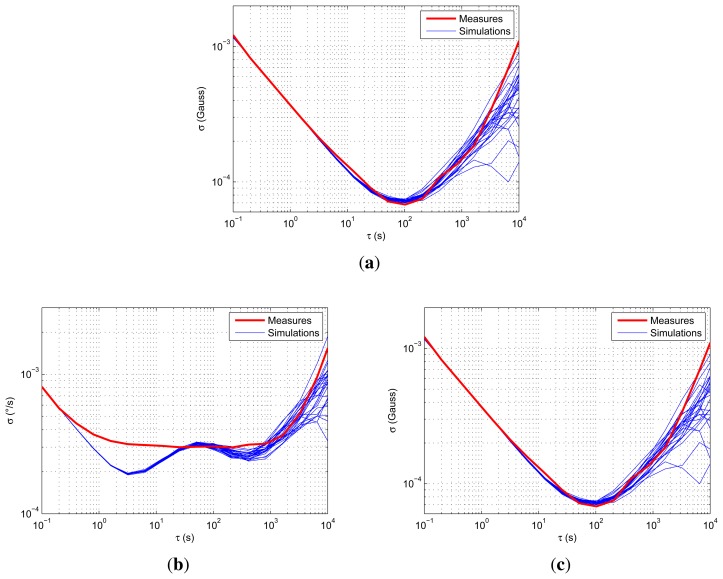
Comparison of Allan variance plots for different simulations and one real measurement. (**a**) Accelerometer; (**b**) rate gyroscope; (**c**) magnetometer.

The simulated motion is a steady position during more than 13 h. It is then simulated 25 times to compare multiple realizations of the stochastic process. The results are illustrated for one magnetometer, one accelerometer and one rate gyroscope in Figure 9.

As can be observed, the rate gyroscopes seem to display high bias instability (the flat part in Figure 9b), but no correlated noise. The simulations surround the measurement curves well, especially for the accelerometers and magnetometers. The latter do not show correlated noise and are mainly composed of white noise (angular random walk slope) and random walk processes (rate random walk slope). As the simulations are close to the real measurements, the error model and the values of the parameters are validated.

## 6. Conclusions and Outlook

In this paper, a magnetometer-augmented IMU simulator has been developed. Its inputs are the position and orientation of the object with respect to the local frame, while its outputs are the sensor measurements. Other parameters can be precisely determined, like the position of the IMU with respect to the object and sensor imperfections. The used sensors are 3D rate gyroscopes, accelerometers and magnetometers. The modeling is based on the quaternion representation of the orientations, avoiding singularities from the Euler angles. Furthermore, the kinematics was used for the determination of the accelerometer measurements. The simulator shows good results during the validation process. It is now operational for estimation algorithm evaluations.

The results obtained with the simulator are compared to the calibrated measurements of a real IMU. The validation is performed by means of an accurate 3D rotational table. The different results prove the accuracy of the simulator, thus validating the equations. As this first validation had low dynamics, another validation process was performed with a gun-launched gyro-stabilized projectile. The results show a good precision and validate the equations.

After the determination and the validation of the error-free measurements, the imperfections were implemented. The typical imperfections modeled here are: sensor dynamics, bias (with a constant part and an evolutive part) and white noise. The parameter identification of the error model was conducted using the Allan variance technique.

The simulator can now be used to simulate different trajectories to test several estimation algorithms. Thanks to all of the details that are included in this simulator, the algorithms can be evaluated by following multiple criteria: the precision of the estimation, the stability regarding long-term navigation, the robustness of the orientation estimator against the accelerations, *etc.* It is also possible to simulate a given trajectory with multiple IMUs performing, then a multi-IMU data fusion, in order to improve the object localization.

## Figures and Tables

**Table 1.** The notations used in this paper.

**Notation Rules**	
*x*	Scalar
***x***	Vector
**X**	Matrix
***x̄*** (or **X̄**)	Transpose of ***x*** (or **X**)
***x̃*** (or **X̃**)	Conjugate of ***x*** (or **X**)

**Kinematic Variables**	

*s*_AB_	Position vector of point A with respect to point B
$\upsilon_{A}^{\text{I}}$	Velocity vector of point A with respect to frame I
$a_{A}^{\text{I}}$	Acceleration vector of point A with respect to frame I
***ω***^EI^	Rotational speed vector of frame E with respect to frame I
***q***^EI^	Quaternion expressing the rotation of frame E with respect to frame I

**Coordinate Systems**	

[***s***_AB_]^E^	Position vector ***s***_AB_ expressed in the coordinate system E
{***q***^EI^}^E^	Quaternion with its vector part expressed in the coordinate system E
[**T**]^EI^	Transition matrix from coordinate system I to coordinate system E

**Table 2.** Numerical results for the standard deviation from the Allan variance applied to the simulation of a low-cost IMU [20]. Here, *unit* represents the unit of the sensor output.

	**Accelerometers**			**Rate Gyroscopes**			**Magnetometers**		
		
	***f***_1_	***f***_2_	***f***_3_	***ω***_1_	***ω***_2_	***ω***_3_	***h*_1_**	***h*_2_**	***h*_3_**
$\sigma_{\text{wn}}\left(\text{unit}/\sqrt{H_{z}}\right)$	3.0*e*^−3^	3.0*e*^−3^	3.0*e*^−3^	2.0*e*^−4^	2.6*e*^−4^	2.2*e*^−4^	3.8*e*^−4^	3.6*e*^−4^	3.7*e*^−4^
$\sigma_{\text{rw}}\left(\text{unit}\cdot \sqrt{H_{z}}\right)$	5.0*e*^−5^	2.7*e*^−5^	4.9*e*^−5^	1.0*e*^−5^	1.6*e*^−5^	1.4*e*^−5^	5.9*e*^−6^	5.1*e*^−6^	8.2*e*^−6^
σ_gm_ (*unit*)	3.5*e*^−3^	0.9*e*^−3^	1.0*e*^−3^	3.0*e*^−4^	5.0*e*^−4^	2.7*e*^−4^	5.9*e*^−5^	2.0*e*^−4^	6.8*e*^−5^
*τ*_gm_(*s*)	300	100	50	50	30	30	50	100	30

**Table 3.** Normalized root mean square error (NRMSE) of the error from all of the different validations. All data are given as the percentage of the full scale.

	**Accelerometers**			**Rate Gyroscopes**			**Magnetometers**		
		
	***f***_1_	***f***_2_	***f***_3_	***ω***_1_	***ω***_2_	***ω***_3_	***h***_1_	***h***_2_	***h***_3_
Test bed validation	1.34	1.66	2.58	0.83	1.51	1.06	3.69	2.03	2.40
Cross simulation validation	0.11	1.08	1.08	4.3*e*^−3^	1.12*e*^−5^	1.28*e*^−5^	NA	NA	NA
